# Anoctamin Calcium-Activated Chloride Channels May Modulate Inhibitory Transmission in the Cerebellar Cortex

**DOI:** 10.1371/journal.pone.0142160

**Published:** 2015-11-11

**Authors:** Weiping Zhang, Steffen Schmelzeisen, Daniel Parthier, Stephan Frings, Frank Möhrlen

**Affiliations:** Department of Animal Molecular Physiology, Centre of Organismal Studies, Im Neuenheimer Feld 504, Heidelberg University, Heidelberg, Germany; Monell Chemical Senses Center, UNITED STATES

## Abstract

Calcium-activated chloride channels of the anoctamin (alias TMEM16) protein family fulfill critical functions in epithelial fluid transport, smooth muscle contraction and sensory signal processing. Little is known, however, about their contribution to information processing in the central nervous system. Here we examined the recent finding that a calcium-dependent chloride conductance impacts on GABAergic synaptic inhibition in Purkinje cells of the cerebellum. We asked whether anoctamin channels may underlie this chloride conductance. We identified two anoctamin channel proteins, ANO1 and ANO2, in the cerebellar cortex. ANO1 was expressed in inhibitory interneurons of the molecular layer and the granule cell layer. Both channels were expressed in Purkinje cells but, while ANO1 appeared to be retained in the cell body, ANO2 was targeted to the dendritic tree. Functional studies confirmed that ANO2 was involved in a calcium-dependent mode of ionic plasticity that reduces the efficacy of GABAergic synapses. ANO2 channels attenuated GABAergic transmission by increasing the postsynaptic chloride concentration, hence reducing the driving force for chloride influx. Our data suggest that ANO2 channels are involved in a Ca^2+^-dependent regulation of synaptic weight in GABAergic inhibition. Thus, in balance with the chloride extrusion mechanism via the co-transporter KCC2, ANO2 appears to regulate ionic plasticity in the cerebellum.

## Introduction

Calcium-activated chloride channels of the anoctamin (alias TMEM16) family of membrane proteins provide a chloride conductance that operates under the control of intracellular Ca^2+^ signals (recent review: [1]). Many different cell types express anoctamin proteins. The anoctamin chloride channels anoctamin 1 (ANO1, TMEM16A) and anoctamin 2 (ANO2, TMEM16B) have been established as Ca^2+^-activated Cl^-^ channels with defined physiological functions [2–4]. They are involved in epithelial Cl^-^ transport, smooth muscle contraction and neuronal signal processing. Anoctamin channels show highly polarized expression patterns in epithelia and neurons, making spatial aspects crucial for understanding channel function. Moreover, ANO1 and ANO2 display a ten-fold difference in Ca^2+^ sensitivity, and various splice forms of these proteins respond to different Ca^2+^ levels [5,6]. Finally, the channels may conduct Cl^-^ influx or Cl^-^ efflux, the balance being decided by the dynamic system of intracellular chloride regulation that includes various Cl^-^/cation co-transporters and their regulatory proteins.

There is a surprising paucity of data on ANO1 and ANO2 in the central nervous system. So far, most of the published data on neuronal expression concern sensory systems. The channels are localized in the chemosensory cilia of olfactory receptor neurons [7–11], in vomeronasal sensory neurons [8,12,13], in rod photoreceptor synaptic terminals [8,14–16], at the cochlear hair-cell synapse, and in the auditory brainstem [17–19], as well as in neurons of the dorsal root ganglia and trigeminal ganglia where they contribute to the processing of heat nociception and inflammatory hyperalgesia [20–25]. Thus, anoctamin chloride channels are clearly involved in the generation, modulation and synaptic transmission of sensory signals. However, based on mRNA expression, there is evidence that the channels are also expressed in various parts of the brain [18,26,27]. But, apart from a proposed epithelial function in the choroid plexus [28] and myogenic effects in cerebral arteries [29,30], there is to our knowledge only one concept for anoctamin-channel function in neuronal networks. In hippocampal neurons, ANO2 appears to influence the efficacy of action potential generation by providing a Ca^2+^-regulated shunt conductance in dendrites, which attenuates output activity [27].

Here we report evidence for a further possible role of anoctamin channels in the brain: the regulation of ionic plasticity of GABAergic synapses in the cerebellar cortex. It was recently reported that cerebellar Purkinje cells use Ca^2+^-activated Cl^-^ channels to modulate the efficacy of synaptic input from inhibitory interneurons, a process termed *depolarization-induced depression of inhibition* (*DDI*) [31]. We carried out expression studies and electrophysiological tests to find out whether the Ca^2+^-activated Cl^-^ channels that mediate *DDI* may be formed by anoctamin proteins. We report that both ANO1 and ANO2 are expressed in the murine cerebellar cortex. The channels display a differential expression pattern. ANO1 is mainly expressed in inhibitory interneurons and in Purkinje cell somata. In contrast, ANO2 is expressed only in Purkinje cells where it is targeted to the dendritic tree. Functional studies revealed that the modulatory effect reported by Satoh *et al*. (2103) [31] is absent in Ano2^-/-^ mice. Our results point to distinct functions of ANO1 and ANO2 in the cerebellar cortex. ANO1 appears to be a component in the network of inhibitory interneurons, while ANO2 may modulate the inhibitory input to Purkinje cells.

## Materials and Methods

### Animals

4 to 20-week-old male C57BL/6 (Black6) mice and Ano2^-/-^ mice [8], kindly provided by Dr. Thomas Jentsch (Leipniz-Institute for Molecular Pharmacology, Berlin), were used for the experiments. GAD^Cre^ mice [32] were kindly provided by Dr. Hannah Monyer (Heidelberg University). Animals were anesthetized and killed by an overdose of isoflurane (Baxter, Germany). All experiments were performed in accordance with the Animal Protection Law and the guidelines and permissions of Heidelberg University.

### Immunohistochemistry

Unfixed fresh mouse brains were embedded in tissue freezing medium (Leica Biosystems) and sliced in a cryotome (Leica CM 3050S). 20 μm thick cryosections were fixed with 2% PFA for 15 minutes, washed 3 × 5 minutes with PBS (130 mM NaCl, 8.1 mM Na_2_HPO_4_, 1.9 mM NaH_2_PO_4_, pH 7.4), and were subsequently processed for staining. DAB-staining was performed using the Vectastain ABC kit guinea pig IgG (biotinylated secondary antibody dilution 1:200; PK-4007; Vector Labs). Sections were incubated in 0.5% H_2_O_2_ for 1 hour and washed 3 × 5 minutes with PBS, followed by incubation in blocking solution (5% normal goat serum, Sigma-Aldrich, G9023, in PBS with 0.5% Triton X-100, 0.05% NaN_3_) for 1 hour. The primary antiserum was diluted in blocking solution and applied overnight at 4°C, then washed for 3 × 10 minutes with PBS. Sections were then incubated in biotinylated secondary antibodies, diluted in blocking solution, for 2 hours, washed 3 × 10 minutes in PBS, incubated in ABC solution (from the ABC Kit, one drop of A and one drop of B in 20 ml PBS, prepared at least 30 minutes before use) for 1 hour, and washed 3 × 10 minutes with PBS. Sections were developed for 5 minutes with DAB-H_2_O_2_ solution (1μl of 1% H_2_O_2_ in 1 ml 3,3'-diaminobenzidin solution; one pellet DAB in 10 ml PBS; Sigma-Aldrich D5905) to start the reaction, washed with PBS intensively, and mounted on glass slides using Aqua-Poly/Mount (Polysciences, Inc., 18606). For immunofluorescence staining, sections were incubated by blocking serum for 1 hour, and the primary antisera (diluted in blocking solution) were applied overnight at 4°C for brains, and 2 hours at room temperature for noses. After washing 3 x 10 min with PBS, the secondary antisera, conjugated with Alexa Fluor tags (Molecular Probes, Inc.), were incubated for 2 hours, washed 3 x 10 minutes with PBS. To visualize cell nuclei, the slices were incubated in DAPI (0.3 μM 4,6-diamidin-2-phenylindol in PBS; Sigma-Aldrich 32670) for 3 minutes, washed 3 x 5 minutes in PBS and mounted on glass slides with Aqua-Poly/Mount. To stain all neurons, we incubated sections in NeuroTrace^®^ 530/615 (Invitrogen N21482, dilution 1:500; Life Technologies, Inc.) for 5 minutes before the DAPI treatment, followed by 3 x 5 minutes wash with PBS. Images were obtained using a Nikon C1 confocal microscope. All imaging data presented are single-plane images, no Z-stacks are used in this paper.

Primary antisera for ANO1 were raised in guinea pig against the intracellular C-terminus (“ANO1_in_^”^ in the text, dilution 1:200, [33]) and in rabbit against the extracellular loop between transmembrane domains 9 and 10 (“ANO1_ex_^”^, dilution 1:250, Alomone Labs, ACL011). Both ANO1 antisera showed the same specific staining patterns in our positive controls (nasal epithelia). The primary antiserum against ANO2 (“ANO2_in_” in the text) was raised in guinea pig against the intracellular loop connecting transmembrane domains 2 and 3 (dilution 1:200; [33]) and was characterized in previous publications [12,26,33]. To verify the specificity of the ANO antisera, we used cryosections of the mouse vomeronasal organ (VNO) because the sensory neurons in the VNO express both ANO1 and ANO2 in their chemosensory microvilli [8,12,13]. All three antisera specifically stained the sensory surface of the VNO. ANO2_in_ immunostaining, but not ANO1 staining, was absent in sections from Ano2^-/-^ mice. Preadsorption with the peptide that was used to raise ANO1_ex_ suppressed the staining of ANO1 but not of ANO2. Cross reactivity of antisera against ANO1 and ANO2 was excluded by staining HEK 293 cells specifically transfected with either channel [33]. Rabbit anti-Cre antiserum (dilution 1:2000) [34] was kindly provided by Dr. Günther Schütz, DKFZ, Heidelberg. Secondary antibodies were goat anti-guinea pig with Alexa Fluor 488 (Molecular Probes A11073, dilution 1:1000) goat anti-guinea pig with Alexa Fluor 568 (Molecular Probes A11075, dilution 1:1000), goat anti-rabbit conjugated with Alexa Fluor 488 (Invitrogen A11008, dilution 1:1000), and goat anti-rabbit conjugated with Alexa Fluor 568 (Invitrogen A11011, dilution 1:1000).

### Expression and molecular characterization of cerebellar Ano1 and Ano2

Semiquantitative RT-PCR analysis was performed on total RNA of cerebellum and, for comparison, on RNA from olfactory epithelium. RNA was isolated using the MagJet RNA Kit (Thermo Scientific). cDNA was synthesized using 5 μg total RNA, oligo (dT) 18 primer, and the Maxima H Minus First Strand cDNA Synthesis Kit (Thermo Scientific). PCR amplification was performed on 1 μl (250 ng) single-stranded cDNA with DreamTaq PCR Master Mix (Thermo Scientific) using the primer pairs ANO1/F861 and ANO2/F865 (Table 1). Cycling conditions were 95°C for 3 min, followed by 28 to 34 cycles at, respectively, 95°C for 30 s, 62°C for 30 s, 72°C for 60 s, and finally 72°C for 8 min. The bands were resolved by gel electrophoresis and were verified by sequencing after purification by GeneJET Gel Extraction Kit (Thermo Scientific). To characterize the splice variants of the Ano1 and Ano2 transcripts, PCR was performed on cerebellar cDNA using the primers listed in Table 1. For Ano1, we used a set of 5 primer pairs which covered the entire transcript according to Genbank Acc. No. NM_178642.5. The forward primer of pair ANO1/F581 matched to the sequence at an alternative predicted translation start site (position 98 in NM_178642.5) and ANO1/F857 matched the sequence at the translation start site of isoform *a* (position 269). ANO1/F855, ANO1/F847 and ANO1/F845 consecutively matched the following sequence of the open reading frame. The primer pair ANO1/F581 resulted in no product while the four other primer pairs resulted in abundant PCR products of predicted size. By sequencing the PCR products, we found that the ANO1*ac* variant is expressed in the cerebellum. For ANO2, the primer pairs ANO2/F871, ANO2/F870 and ANO2/F869 consecutively matched the sequences of three alternative predicted translation start sites according to Genbank Acc. No. NM_153589.2. ANO2/F867, while ANO2/F905 matched the remaining sequence of the open reading frame. PCR products of predicted size were obtained only for primer pairs ANO2/F869, ANO2/F867 and ANO2/F905, indicating that the first two alternative translation start sites are not present in the cerebellar transcript. By sequencing all PCR products, we found that the cerebellar ANO2 isoform is identical to the main olfactory isoform [6,9].

**Table 1:** PCR primer pairs used to characterize cerebellar ANO1 and ANO2.

**Semiquantitative RT-PCR**		
ANO1/F861	Forward	GGCCCGGTGACTACGTGTA
	Reverse	GCTGTGCCATTCTGGAAGTCGC
ANO2/F865	Forward	TTGAGATTGGAGTCCCGAAGCTAA
	Reverse	GGTGCCCATGGTGGTTCTCGATAA
**Characterization of the splice variants**		
ANO1/F851	Forward	ATGCAGGACGCGCAGGACA
	Reverse	GACTCCGTAACTTGCCCATTCC
ANO1/F855	Forward	CGCAGCGTCCACATCGTGAAC
	Reverse	ACTTGCCCATTCCTCATACAG
ANO1/F857	Forward	ATGAGGGTCCCCGAGAAGTACTC
	Reverse	CAGGAGGCCTCGCGTCTCA
ANO1/F847	Forward	CCCGGGAGAAGCAACACCTATT
	Reverse	AGTCACCGGGCCGACCAAC
ANO1/F845	Forward	CGGTGACTACGTGTACATCTTCC
	Reverse	GGCCTGTCATCATGGCTACAG
ANO2/F871	Forward	AGAGGAAGCCAGGGCCCCAAAC
	Reverse	CCGCTTCAGAATCTCGTGTAC
ANO2/F870	Forward	TGGGCTGCGAGACATCC
	Reverse	CCGCTTCAGAATCTCGTGTAC
ANO2/F869	Forward	CGCATGCACTTTCACGACAAC
	Reverse	CCGCTTCAGAATCTCGTGTAC
ANO2/F867	Forward	CCCCACCAAGAAAATGTACGAG
	Reverse	GGTAACCGTCGAACACGTAGACA
ANO2/R905	Forward	CCCTGACGTTCTCCATTGTC
	Reverse	CGGGCTCATACGTTGGTGTG
**Full lenght cloning**		
ANO1/F524	Forward	AGAATTCCACCATGAGGGTCCCCGAGAAGTACTC
	Reverse	TGGATCCAACAGCGCGTCCCCATGGTAC

### Immunoblot analysis

Main olfactory epithelium, eyes, olfactory bulb and cerebellar tissue were dissected from wild-type C57BL/6 (Black6) or Ano2^-/-^ mice. A whole-protein extraction method was used for most of the samples. For biochemical assays of ANO2 expression in olfactory bulb and cerebellum, the Qproteome Cell Compartment Kit (Qiagen) was used for protein extraction because it was reported to produce particularly high yield of membrane protein [35]. The supernatant of fraction 2, containing primarily membrane proteins, was separated by SDS–PAGE on 10% gels and electro-blotted to PVDF membranes (Machery & Nagel; Germany) using a semidry blotting apparatus. Membranes were blocked with 5% milk powder (in PBS / 0.1% Tween 20) for 1 hour and incubated with the primary antibodies overnight. The blots were washed three times with 0.1% Tween 20 in PBS and incubated for 1 hour with a horseradish peroxidase-conjugated secondary antibody. The blots were washed again, and the *ECL plus* enhanced chemoluminescence system (GE Healthcare, Germany) was used to monitor bound antibodies. Antibodies used for immunoblotting were guinea-pig anti-ANO1 (dilution 1:400; C-terminus encoding amino acids 962–1040; marked "ANO1_in_" in the text) [33], and a rabbit ANO2 antiserum directed against the extracellular loop that connects TMDs 5 and 6 in ANO2 (Alomone Labs, ACL012, dilution 1:1000, marked “ANO2_ex_” in the text). The specificity of the ANO2_ex_ antiserum was verified using olfactory epithelium and VNO, comparing wild-type and ANO2^-/-^ mice. Furthermore, we used goat anti-actin (Santa Cruz sc-1615, dilution 1:1000), rabbit anti guinea pig HRP secondary antibody (Sigma, dilution 1:20000), goat anti rabbit HRP secondary antibody (Sigma, dilution 1:30000), donkey anti goat HRP secondary antibody (Jackson ImmunoResearch, dilution 1:10000).

### EYFP-tagged Ano1 and Ano2 expression plasmids

For heterologous expression in HEK 293 cells, EYFP-tagged mouse cerebellar ANO1 and ANO2 were used. For ANO1*ac*, the primer pair ANO1/F524 (Table 1) was used for full length cloning from cerebellar cDNA. For controls, the ANO1*abc* isoform was amplified from mouse Ano1*abc* pCMV-SPORT6 plasmid (kindly provided by Dr. Rainer Schreiber und Dr. Karl Kunzelmann, University of Regensburg) using the same primers. An *EcoR*I site was introduced at the 5’end and a *BamH*I site at the 3’ end of the fragments for fusing EYFP to the C-terminus in the expression vector pEYFP-N1 (Takara Bio Europe/Clontech, France). The ANO2-pEYFP-N1 expression plasmid was kindly provided by Dr. Johannes Reisert (Monell Chemical Senses Center, Philadelphia).

### Electrophysiology

The cerebellum was removed from the skull directly after sacrificing the animals, and was mounted with cyanoacrylate glue in a vibratome chamber (Leica VT1000S) filled with the respective artificial cerebrospinal fluid (ACSF) at 34–37°C. Sagittal tissue slices were cut at 220–250 μm and transferred to an incubation chamber filled with ACSF at 34^°^C for 30 min. After this, the incubation chamber was kept at room temperature. All solutions were saturated with 95% O_2_ / 5% CO_2_ [36]. For patch-clamp experiments, we transferred the tissue slices onto the stage of an upright, water immersion microscope (Nikon Eclipse E600FN). The recording chamber was continuously perfused with oxygenated ACSF at room temperature. Visual control was achieved with a camera system (Nikon DN100), and Purkinje cells were identified by their shape and location. To obtain whole-cell recordings, borosilicate glass micropipettes (Science Products, Hofheim, Germany; GB-150F-10) were made using a horizontal puller (Sutter Instruments; P97) to a resistance of 2–3 MΩ. Pipettes were filled with an intracellular solution and positioned on the Purkinje cell soma. After obtaining a tight seal, the plasma membrane inside the pipette tip was disrupted by suction to establish the whole-cell configuration. The drug perfusion system operated at 1–2 ml/min for a complete exchange of the bath solution. To block presynaptic cannabinoid receptors, which tend to reduce inhibitory signals [37], the CB1 antagonist *AM251* (2 μM) was included in the bath solution. Electrical signals were recorded using a patch-clamp amplifier (HEKA Electronics, Lambrecht, Germany; EPC-8) and the WinWCP (4.6.1) program provided by the University of Strathclyde, Glasgow, UK. Capacitance compensation was applied; series resistance was not compensated. Holding voltages were corrected for liquid junction potentials. Stimulation of climbing fibers was triggered by a constant current source (Digitimer, Welwyn Garden, UK; DS7A) through an ACSF-filled pipette. Postsynaptic currents were counted off-line before and after stimulation of climbing fibers. 3-minute traces were scanned by a peak-detection software (Mini-Analysis Program, Synaptosoft Inc.). Further analysis was performed using Igor Pro (WaveMetrics, version 6.34 A).

The following three intracellular solutions were used for Purkinje-cell electrophysiology: (1) for 131 mM [Cl^-^]_i_ (mM): 119 CsCl, 6 MgCl_2_, 10 EGTA, 10 HEPES, 4 Na_2_ATP, 19 sucrose; pH 7.3 with CsOH; (2) for 12 mM [Cl^-^]_i_ (mM): 119 Cs-methane sulfonate, 6 MgCl_2_, 10 EGTA, 10 HEPES, 4 Na_2_ATP, 19 sucrose; pH 7.3 with CsOH; (3) for 5 mM [Cl^-^]_i_ (mM): 150 Cs-methane sulfonate, 5 KCl, 0.1 Cs-EGTA, 10 Na-HEPES, 3 Mg-ATP, 0.4 Na-GTP; pH 7.4 with CsOH. ACSF for 131 mM [Cl^-^]_i_ experiments and 12 mM [Cl^-^]_i_ experiments (mM): 119 NaCl, 26.2 NaHCO_3_, 2.5 KCl, 1 NaH_2_PO_4_, 1.3 MgCl_2_, 2.5 CaCl_2_, 33 glucose; pH 7.4 with HCl. ACSF for 5 mM [Cl^-^]_i_ experiments (mM): 138.6 NaCl, 21 NaHCO_3_, 3.4 KCl, 0.6 NaH_2_PO_4_, 1 MgCl_2_, 2.5 CaCl_2_, 15 glucose; pH 7.4 with HCl. Drugs used were the ANO1-inhibitor *T16Ainh-A01* (Tocris Bioscience, 4538), the GABA_A_ channel blocker picrotoxin (Sigma-Aldrich, P1675), and the cannabinoid receptor 1 antagonist *AM251* (Tocris Bioscience, 1117). Alexa Fluor 568 hydrazide sodium salt (Molecular Probes, A-10437, 50 μM) was used to visualize Purkinje cells.

For heterologous expression, EYFP-tagged mouse cerebellar Ano1 or Ano2 was transfected into HEK 293 cells by Ca^2+^-phosphate co-precipitation. 24 h after transfection, expression was confirmed by yellow fluorescence, and cells were examined in whole-cell configuration at -70 mV. The bath solution contained (mM): 150 CsCl, 10 HEPES, 10 EGTA; pH 7.4 (CsOH); the pipette solutions contained 133.5 mM CsCl, 8.26 mM CaCl_2_, 10 mM HEDTA, 10 mM HEPES; pH 7.0 (CsOH), to give a free Ca^*2+*^ concentration of 7.5 μM [38]. Currents were recorded immediately after whole-cell break-through as Ca^*2+*^ diffused into the cell. To evaluate the data for ANO2, the maximal current amplitudes were related to the individual cell capacitance. The current densities from 19–30 cells were averaged for each test. With ANO1 expressing cells, we did not obtain individual values for cell capacitance because large chloride conductances prevented accurate determination. In this case, current densities were calculated using the average cell capacitance of ANO2-expressing HEK 293 cells in our experiments. To test the effect of *T16Ainh-A01*, 5 μM of the compound were continuously included in the bath solution. Statistical analysis was done using Student´s *t* test. Error bars indicate SEM; significance levels were p < 0.05 (*), p < 0.01 (**), p < 0.001 (***) or *p*< 0.0001 (****).

## Results

### Detection of ANO1 and ANO2 expression in the mouse cerebellum

**Fig 1:**
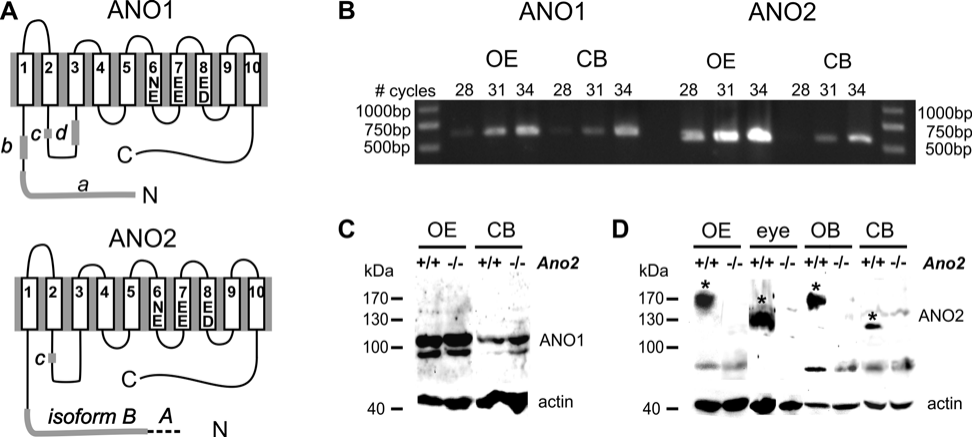
ANO1 and ANO2 expression levels in the cerebellum. **(A)** Membrane topology model for anoctamin Ca^2+^-activated Cl^-^ channels based on the X-ray structure of a fungal TMEM16 protein [42]. The transmembrane domains 5 and 6 are thought to provide the pore-lining region in the homodimeric channel [95]. Five negatively charged amino-acid residues *(E*, *D)* and an asparagine residue *(N)* in transmembrane domains 6–8 serve as Ca^2+^-binding sites involved in channel gating [39–41]. Four alternatively spliced segments *(a—d)* determine the apparent Ca^2+^-sensitivity of the ANO1 channel [5]. ANO2 has two isoforms *A* and *B* and a regulatory motif at a position homologous to segment *c* in ANO1 [6]. **(B)** RT-PCR analysis from mouse olfactory epithelium *(OE)* and mouse cerebellum *(CB)* yield similarly strong ANO1 signals in cerebellum but weaker signals for ANO2. **(C)** Immunoblots obtained from lysates of cerebellum *(CB)* and main olfactory epithelium *(OE)* from wild-type and Ano2^-/-^ mice show an ANO1-specific signal at ~120 kDa with the ANO1_in_ antiserum. **(D)** Rabbit anti-ANO2_ex_ serum stains ANO2-specific bands *(asterisks)* in immunoblots obtained from lysates of main olfactory epithelium *(OE)* and eye, as well as in membrane-protein preparations of main olfactory bulb *(OB)* and cerebellum *(CB)*. ANO2 bands are not present in immunoblots from Ano2^-/-^ mice.

ANO1 and ANO2 channels are thought to have similar membrane topology. Hydropathy analyses and various functional assays have pointed to a model with 8 transmembrane domains [39–41] which was refined by X-ray crystallography to a 10 TMD model (Fig 1A) [42]. We compared the cerebellar expression levels of both genes with the olfactory neuroepithelium because the expression of both proteins is well characterized in that tissue [33]. The PCR signal for ANO1 cDNA was comparable between cerebellum and olfactory epithelium, while the ANO2 cDNA was weaker in cerebellum than in olfactory epithelium (Fig 1B). Both for ANO1 channels and for ANO2 channels, several alternative translation forms exist [5,6,41]. For ANO1, the positions of four relevant exons are indicated as *a—d* in Fig 1A. To identify the cerebellar ANO1 isoform, we used a set of 5 overlapping primer pairs (Table 1) that, together, covered the entire open reading frame of the transcript. By sequencing the PCR products, we found that the ANO1*ac* variant is expressed in the cerebellum. This isoform was previously shown to encode Cl^-^ channels with particularly high apparent Ca^2+^ sensitivity (K_D_ = 0.15 μM at -40 mV; [5]). ANO2 proteins exist in the two isoforms *A* and *B* in olfactory receptor neurons [6] (Fig 1A). By sequencing the PCR products from cerebellum, we found the isoform *B* to be the predominant ANO2 variant. Isoform *B* is also the prevalent form in olfactory receptor neurons. It may contain a regulatory motif at a position homologous to segment *c* of ANO 1. This motif is present in photoreceptors but not in olfactory receptor neurons [26]. We found that it is also absent from the cerebellar ANO2 sequence. In immunoblots from membrane-protein preparations, the ANO1_in_ serum labeled a band at the expected size of 115 kDa in olfactory epithelium and in cerebellum, both in wild-type and in Ano2^-/-^ mice (Fig 1C). A weaker second band was visible at ~95 kDa. For detection of ANO2 protein, we used the ANO2_ex_ antiserum which labeled a discrete band in cerebellum, as well as broader bands in preparations from olfactory epithelium, olfactory bulb and eye, all of which were absent in Ano2^-/-^ mice (Fig 1D). These signals are characteristic for the glycosylated ANO2 protein at ~120 kDa in eye and at 150–170 kDa in olfactory tissues [8]. In our membrane-protein preparation, the cerebellar ANO2 protein appeared at ~120 kD with no evidence for pronounced glycosylation. These data consistently demonstrate that ANO1 and ANO2 proteins are expressed in the mouse cerebellum.

### Differential expression of ANO1 and ANO2 in the cerebellar cortex

**Fig 2:**
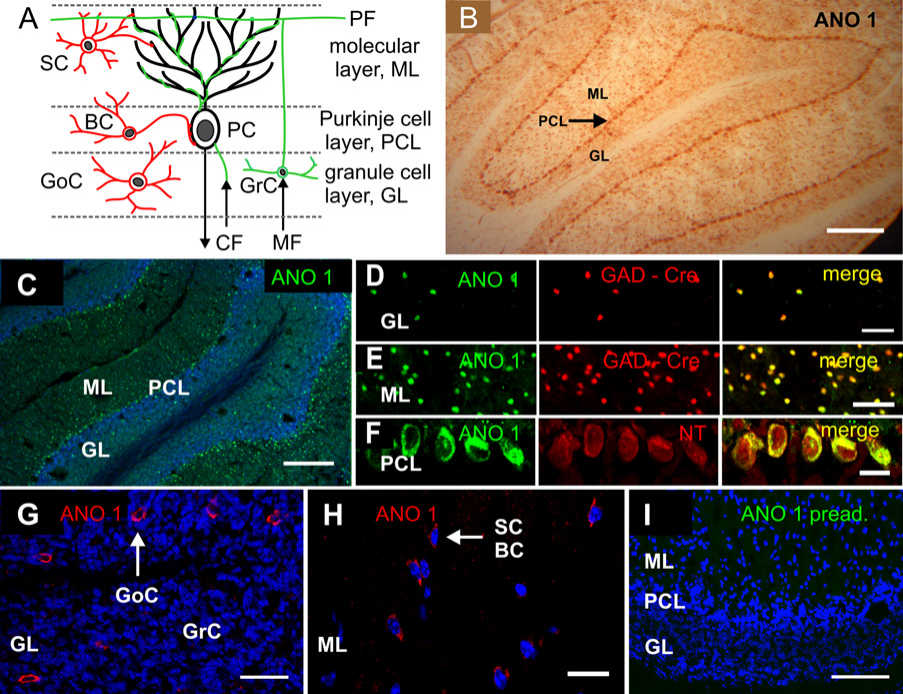
Expression of ANO1 in neurons of the cerebellar cortex. **(A)** Schematic representation of the main cell types that constitute the microcircuits of the cerebellar cortex. Inhibitory interneurons *(red)*: basket cells *(BC)*, Golgi cells *(GC)* and stellate cells (SC); excitatory input to Purkinje cells *(PC) (green)*: climbing fibers *(CF)*, granule cells *(GrC)*, mossy fibers *(MF)* and parallel fibers *(PF)*. **(B)** Diaminobenzidine-labelled ANO1_in_ antiserum produced a continuous staining pattern in the Purkinje cell layer *(PCL)* as well as a scattered stain in the granule cell layer *(GL)* and the molecular layer *(ML)* of the mouse cerebellum. **(C)** Low-magnification image showing ANO1 immunofluorescence *(green)* and DAPI nuclear stain *(blue)*. Only few ANO1-positive cells are located among the many cells of the *GL*, most ANO-1 cells can be seen in the *PCL* and *ML*. **(D)** Detail of the *GL*, demonstrating co-localization of ANO1 and GAD^Cre^, a marker for GABAergic neurons. **(E)** Co-localization of ANO1 with GAD^Cre^ cells in the *ML*. **(F)** Purkinje cell somata stained with the ANO1_ex_ antiserum and *NeuroTrace* ^®^ to illustrate that all Purkinje cells are ANO1-positive. **(G)** Small, ANO1-negative granule-cell nuclei *(blue)* and somewhat larger ANO1-positive Golgi cells *(GoC)* in the *GL*. **(H)** In the molecular layer, ANO-1 positive cells are *SC* and *BC* inhibitory interneurons. **(I)** Preadsorption control for the ANO1_ex_ antiserum on cerebellar cortex. Scale bars: *B*, *C*: 200 μm, *D*,*E*: 50 μm, *F*: 20 μm, *G*: 50 μm, *H*: 20 μm, *I*: 50 μM. Blue represents DAPI nuclear stain.

Purkinje cells, the large output neurons of the cerebellar cortex, receive excitatory input from granule cells and climbing fibers, as well as inhibitory input from stellate cells, basket cells and Golgi cells (Fig 2A). To find out which cells express ANO1 and ANO2, cryosections were prepared without pre-fixation of the tissue. DAB-labeled ANO1_in_ antibodies stained the Purkinje cell layer as well as scattered cells in the molecular layer and granule cell layer (Fig 2B and 2C). Inhibitory interneurons in the granule cell layer (Fig 2D) and in the molecular layer (Fig 2E) were immunopositive both for ANO1 and for glutamate decarboxylase (GAD), a marker for GABAergic neurons. We used a mouse line that expressed Cre recombinase in all GAD67-expressing cells [32], and immunostained with an antiserum raised against Cre recombinase [34]. The labeled neurons in the granule cell layer are probably Golgi cells (Fig 2G) while the GAD/ANO1-positive cells in the molecular layer are basket/stellate cells (Fig 2H). Thus, apparently all GABAergic cells in the cerebellar cortex were ANO1-positive. The somata of Purkinje cells also expressed ANO1 (Fig 2F). Co-staining with the neuronal marker *NeuroTrace* ^*®*^ revealed that *all* Purkinje cells contained ANO1. However, ANO1 immunosignals were faint in, or absent from, the Purkinje cell dendrites. The preadsorption control with the immunizing peptide showed no immunosignal (Fig 2I).

**Fig 3:**
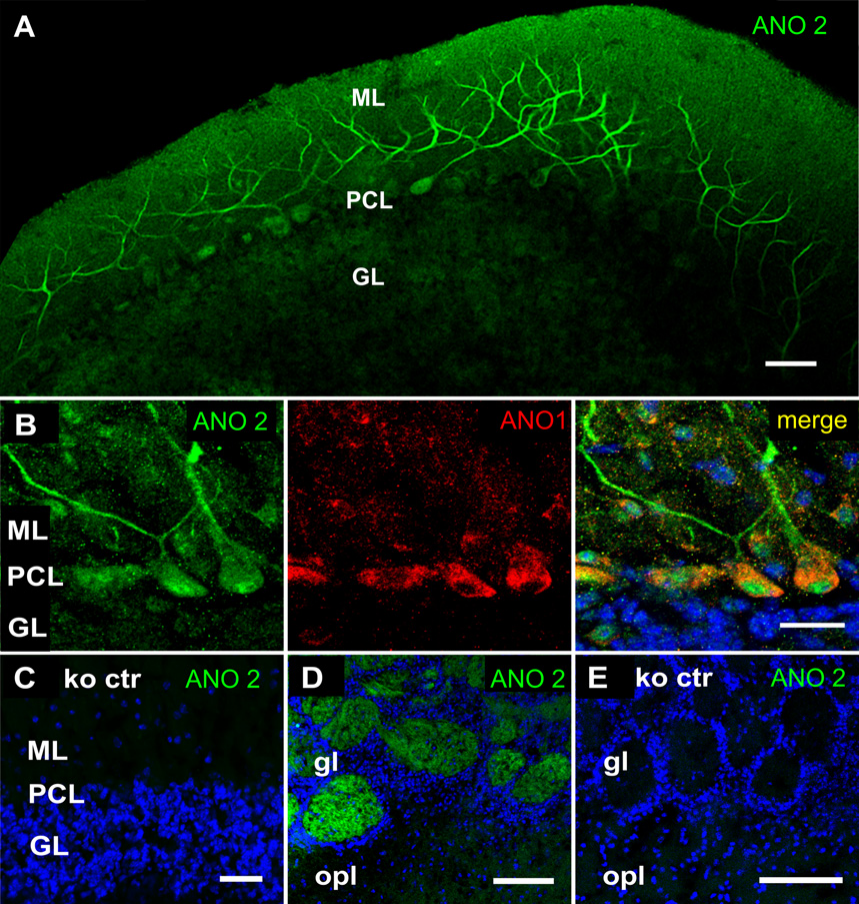
Expression of ANO2 protein in Purkinje cells. **(A)** ANO2 immunosignals from the cerebellar cortex are discernible in the dendrites of Purkinje cells. The signals are weak but stronger than the background signals emanating from the granule cell layer *(GL)*. **(B)** In the Purkinje cell, the ANO2 immunosignal *(green)* is visible in dendrites and the perinuclear region, but only weakly in the plasma membrane of the cell body. In contrast, ANO1 signals *(red)* label the entire Purkinje cell soma, but are not detectable in dendrites. **(C)** The ANO2 antiserum does not stain the cerebellar cortex of the Ano2^-/-^ mouse. **(D)** ANO2 immunosignals in the glomeruli of the olfactory bulb serving as positive control for ANO2 in brain tissue; *gl*: glomerular layer, *opl*: outer plexiform layer. **(E)** Absence of ANO2 immunosignals from the olfactory bulb of the Ano2^-/-^ mouse. Blue in *B-E* represents DAPI nuclear stain. All calibrations bars: 20 μm.

Immunostaining of cerebellar cortex cryosections with ANO2_in_ antiserum revealed a different expression pattern compared to ANO1. Like ANO1, the ANO2 immunosignal was absent from granule cells. However, ANO2 was also absent from the inhibitory interneurons. Instead, the protein was located in the Purkinje-cell dendrites (Fig 3A). Within the somata of Purkinje-cells, ANO2 immunosignals appeared to be confined to the perinuclear area, presumably the rough endoplasmic reticulum. This contrasts with ANO1 expression, as ANO1 protein can be detected all through the soma, but not in the dendrites (Fig 3B). For ANO2, a viable knockout mouse line is available [8]. To test the specificity of the cerebellar ANO2 signal, we stained cryosections of Ano2^-/-^ mice and detected no immunosignals under the same experimental conditions (Fig 3C). The only other structure in the brain reported to be immunopositive for ANO2 is the olfactory bulb where the axons of olfactory receptor neurons coalesce onto glomeruli to form their synapses [8]. Fig 3D and Fig 3E depict the ANO2 immunosignal in olfactory bulb as a positive control and its absence from the Ano2^-/-^ mouse.

These data reveal a differential expression pattern of ANO1 and ANO2 in the cerebellar cortex. Only ANO2 is present at detectable density in the dendrites, the site of synaptic plasticity of inhibitory transmission as discovered by Satoh et al. (2013) [31]. We, therefore, asked whether ANO2 may be involved in this process.

### Plasticity of GABAergic inhibition is altered in Ano2^-/-^ mice

**Fig 4:**
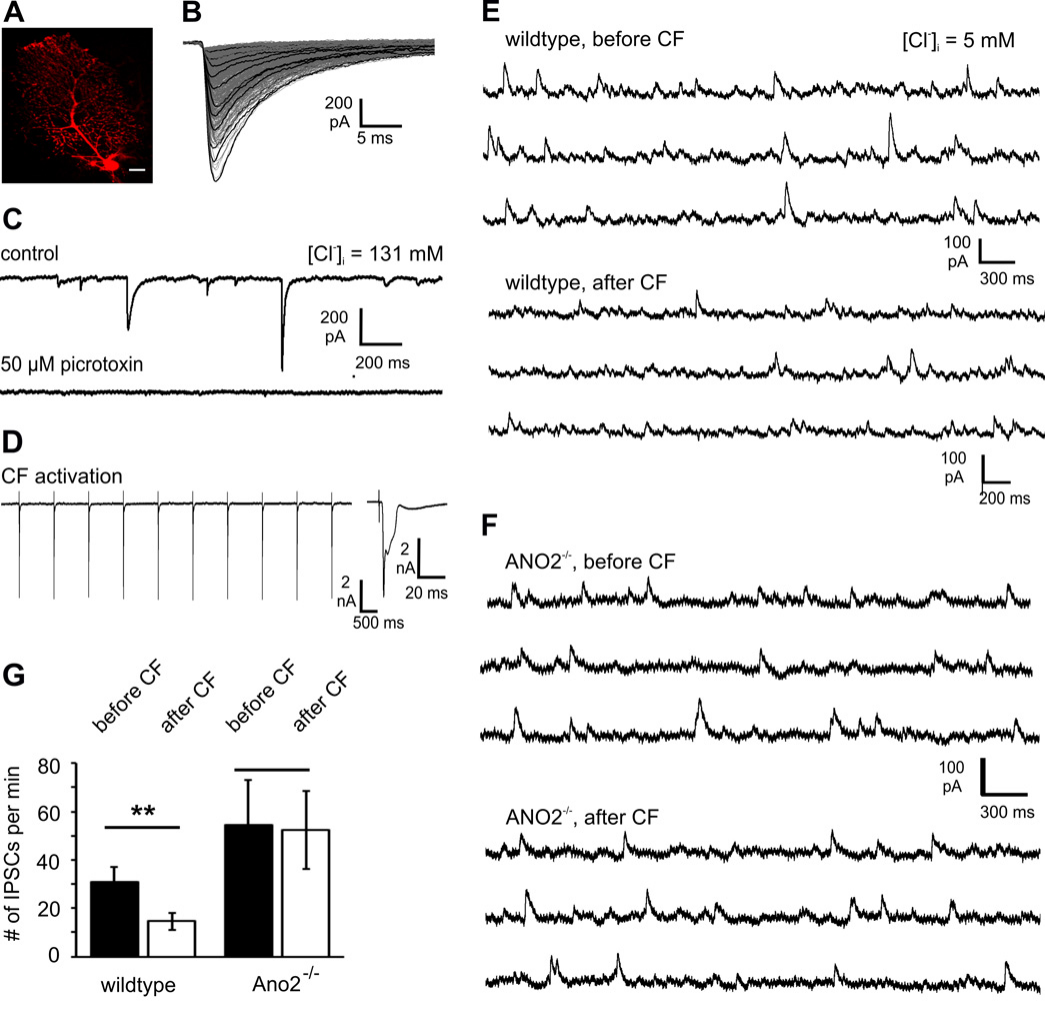
Involvement of ANO2 in depolarization-induced depression of inhibition. **(A)** A cerebellar Purkinje cell loaded with the fluorescent dye Alexa Fluor 568. Scale bar: 10 μm. **(B)** Spontaneous postsynaptic currents in a Purkinje cell with E_Cl_ near 0 mV and V_hold_ = -69 mV. Overlay of 764 current traces showing similar time courses but differing amplitudes, probably reflecting distinct positions of GABAergic synapses on the Purkinje cell dendritic tree. **(C)** Postsynaptic currents were completely blocked by 50 μM picrotoxin, an inhibitor of GABA_A_-receptor chloride channels. **(D)** Protocol for activation of climbing fibers: Ten 0.1-ms current pulses were applied to the area near the proximal dendrite of a Purkinje cell while recording the whole-cell current of that cell at -70 mV. CF-activation produced characteristic complex spikes, as shown in the inset. **(E)** *Upper traces*: GABAergic inhibitory postsynaptic currents recorded from a Purkinje cell at V_hold_ = -48 mV and with 5 mM Cl^-^ in the pipette solution. The positive polarity of IPSCs indicates Cl^-^ influx. *Lower traces*: postsynaptic currents, recorded immediately after the climbing-fiber stimulation, displayed decreased amplitudes. **(F)** IPSCs recorded from a Purkinje cell of an Ano2^-/—^mouse before *(upper traces)* and immediately after *(lower traces)* CF-activation. **(G)** The number of detectable IPSC signals decreased by ~47% through climbing-fiber stimulation (before CF: 30.7 ± 6.5 min^-1^; after CF: 14.6 ± 3.4 min^-1^; 8 cells; *ctrl*). In slices from Ano2^-/-^ mice, more IPSCs were detected (54.5 ± 18.5 min^-1^; 4 cells), and the activation of climbing fibers had no effect (52.5 ± 16.2 min^-1^; 4 cells).

Satoh et al. (2013) [31] found that Ca^2+^-dependent Cl^-^ currents were activated in Purkinje cells during stimulation of climbing fibers, and that these Cl^-^ currents caused a depression of GABAergic IPSCs recorded from Purkinje cells. To find out whether ANO2 is involved in this process, we compared the effect of climbing-fiber stimulation on IPSCs in wild type and ANO2^-/-^ mice. Whole-cell recordings were obtained from sagittal tissue slices of mouse cerebellum. Purkinje cells located underneath the surface of the slice were identified by positions and shapes of their cell bodies and were selected for viability by their smooth, convex plasma membranes. A fluorescent dye was occasionally included in the pipette solution to visualize the dendritic tree (Fig 4A). Postsynaptic currents were recorded at a holding voltage V_hold_ of -69 mV and an intracellular Cl^-^ concentration [Cl^-^]_i_ of 131 mM. Detectable postsynaptic currents varied from a few pA to 819 pA (mean: 200.6 ± 5.8 pA). Their time course was characterized by a mean rise time (interval from 10% to 90% of maximal current) of 0.75 ± 0.007 ms, and a mean decay time constant (single-exponential fit) of τ = 5.54 ± 0.06 ms (Fig 4B; overlay of 764 signals). The signals were completely blocked by 50 μM picrotoxin in the bath solution (Fig 4C) and were thus pharmacologically identified as GABAergic currents. They originated from synapses that inhibitory interneurons (basket cells, stellate cells) form on the Purkinje cell dendrites and somata [43]. The negative polarity of these signals indicates that GABA_A_ receptors conduct Cl^-^ efflux at the high intracellular chloride concentration used. In these experiments, differences in amplitude may, in part, result from different local [Cl^-^]_i_ levels at each individual synapse. Moreover, electrical signals caused by dendritic postsynaptic currents in Purkinje cells are considerably attenuated and filtered as they travel along the dendrite toward the soma. With increasing distance between soma and the synaptic location on the dendritic tree, signal amplitudes decrease and time constants increase [44].

ANO2 channels require more than 1 μM Ca^2+^ for full activation [9,11,38]. Dendritic Ca^2+^ influx was generated through synaptic activity of climbing fibers (CF) [45–47], which was triggered near the proximal dendrites of Purkinje cells through a stimulation pipette. CF activation designed to produce *DDI* (20 pulses at 1 Hz; Fig 4D) was confirmed by recording from the Purkinje cell the characteristic, prolonged Ca^2+^ currents that underlie complex-spike formation [48]. [Cl^-^]_i_ was set at 5 mM in these experiments (E_Cl_ = -88 mV) to obtain inhibitory postsynaptic currents from the GABAergic synapses. At V_hold_ between -60 mV and -45 mV, the synaptic signals were positive, indicative of postsynaptic Cl^-^ influx under this low-chloride condition (Fig 4E, *upper panel*). Following climbing-fiber stimulation, the recordings contained IPSC signal with reduced amplitudes (Fig 4E, *lower panel*). According to the concept of *DDI* [31], we interpret this reduced amplitude as indicative of a reduced Cl^-^ driving force for GABA-induced currents (see Discussion). To quantify the effect, we counted all IPSCs that were detected over 3 minutes. In ANO2^+/+^ mice, the number of these signals decreased by 47% (8 cells) following climbing fiber stimulation (Fig 4G). The same experiments were carried out with cerebellar slices from ANO2^-/-^ mice and revealed two differences compared to the wildtype: The number of IPSCs per minute before climbing-fiber activation was almost 2-fold higher, and it was not affected by climbing-fiber activation (Fig 4G). This observation is a strong indication for a role of ANO2 in the Ca^2+^-dependent depression of inhibition described by Satoh et al. (2013) [31].

### An ANO2 channel inhibitor mimics the effect of the ANO2 knockout

**Fig 5:**
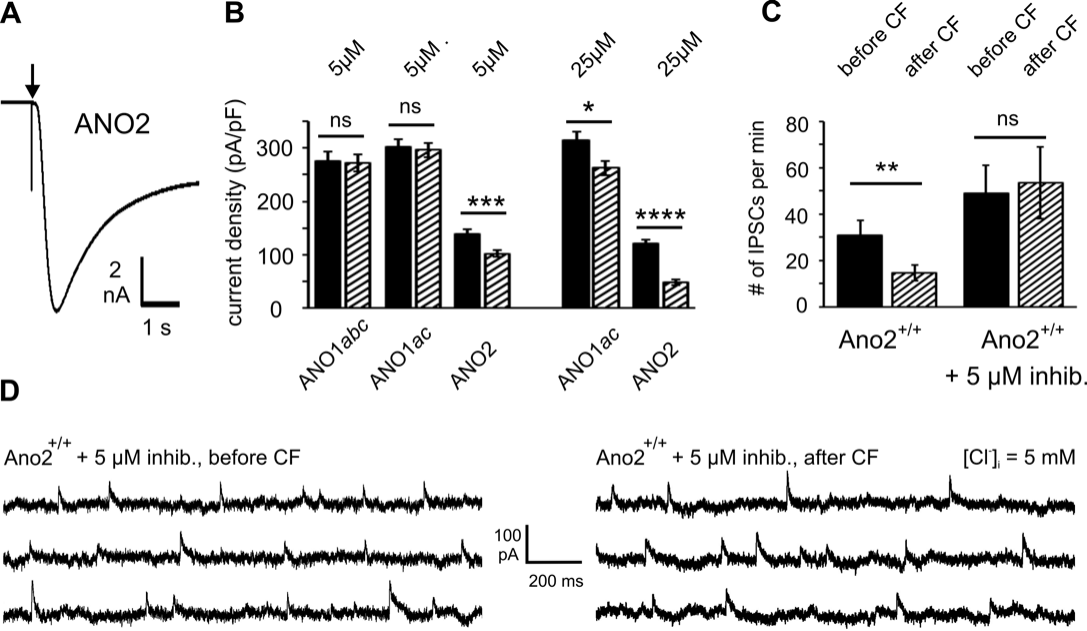
Identification of an inhibitor specific for cerebellar ANO2 channels. **(A)** Whole-cell recording from an HEK293 cell transfected with mouse cerebellar ANO2 isoform *B* at -70 mV. Chloride inward current was activated by diffusion of 7.5 μM Ca^2+^ from the pipette into the cell immediately after whole-cell breakthrough *(arrow)*. **(B)** Results from current recordings without *(black bars)* and with *(hatched bars) T16Ainh-A01* revealed that neither mouse ANO1*ac* nor ANO1*abc* were significantly inhibited by 5 μM of the compound, while the ANO2 isoform *B* showed a significantly reduced current density. At 25 μM *T16Ainh-A01*, significant inhibition was also observed with ANO1*ac*. Results were averaged from 19–30 cells for each condition. **(C)** When applied at the ANO2-specific concentration of 5 μM, *T16Ainh-A01* blocked *DDI*: The number of detectable IPSCs was 48.9 ± 12.1 min^-1^ before and 53.4 ± 15.4 min^-1^ after CF stimulation. ANO2^+/+^ control data from Fig 4G are included for comparison. **(D)** IPSC traces from wildtype mice in the presence of 5 μM *T16Ainh-A01* show no difference in IPSC shapes and amplitudes before *(left traces)* and after *(right traces)* CF-activation.

A reversible ANO2 channel inhibitor would be helpful to further investigate the physiological function of ANO2 in Purkinje cells. The widely used ANO1/ANO2 blocker niflumic acid was not suitable for our experiments because it is not specific for ANO2, and it is also known to have a potentiating effect on GABA_A_ receptors [49]. The channel inhibitor *T16Ainh-A01* was originally identified as an ANO1 inhibitor in a small-molecule screen [50] and characterized in various cell types [50–52]. The efficacy of this compound in blocking anoctamin channels depends on the animal species and on the splice variant of the channels. In particular, *T16Ainh-A01* does not effectively inhibit mouse ANO1 [52], but strongly inhibits ANO2 channels [50]. To test its suitability for our experiments on cerebellar ANO2 channels, we expressed the cerebellar variants of murine ANO1 and ANO2 in HEK293 cells and examined their sensitivity to blockage by *T16Ainh-A01*. Transfected HEK 293 cells were perfused with pipette solution containing 7.5 μM Ca^2+^, and the resulting currents were recorded at -70 mV immediately after whole-cell breakthrough (Fig 5A). This whole-cell method of channel blockage was used because it avoids channel alterations due to run-down, which ANO1 and ANO2 exhibit upon excision from the cell [26]. The average current densities were determined without inhibitor and compared to the values obtained in the presence of 5 μM or 25 μM *T16Ainh-A01*. In accordance with the report by Namkung et al. (2011) [50], the compound displayed a clear selectivity for ANO2 over ANO1 (Fig 5B). The compound did not affect currents conducted by mouse ANO1 channels at 5 μM, neither in the *abc* nor in the *ac* splice variant. Only at 25 μM *T16Ainh-A01* were ANO1*ac* channels significantly inhibited. In contrast, ANO2-mediated currents were reduced by 27% at 5 μM and by 60% at 25 μM. As 5 μM *T16Ainh-A01* selectively inhibits cerebellar ANO2 channels, we used this concentration to isolate pharmacologically any contribution of these channels to Purkinje cell activity. Although 5 μM *T16Ainh-A01* exerts only a moderate inhibitory effect on ANO2, it is suitable to experimentally distinguish ANO2 effects from any contributions by ANO1. In cerebellum slices incubated in ACSF containing 5 μM of the inhibitor, we recorded significantly increased IPSC numbers per minute. This elevated IPSC frequency was resistant to stimulation of climbing fibers (Fig 5C and 5D). In fact, the IPSC activities before and after the induction of complex spikes did not differ significantly between Ano2^-/-^ mice and wild-type mice recorded with 5 μM channel inhibitor. These results further support the hypothesis that ANO2 mediates the plasticity of GABAergic inhibition in Purkinje cells.

### Ionic plasticity may underlie the ANO2 effect on GABAergic inhibition

**Fig 6:**
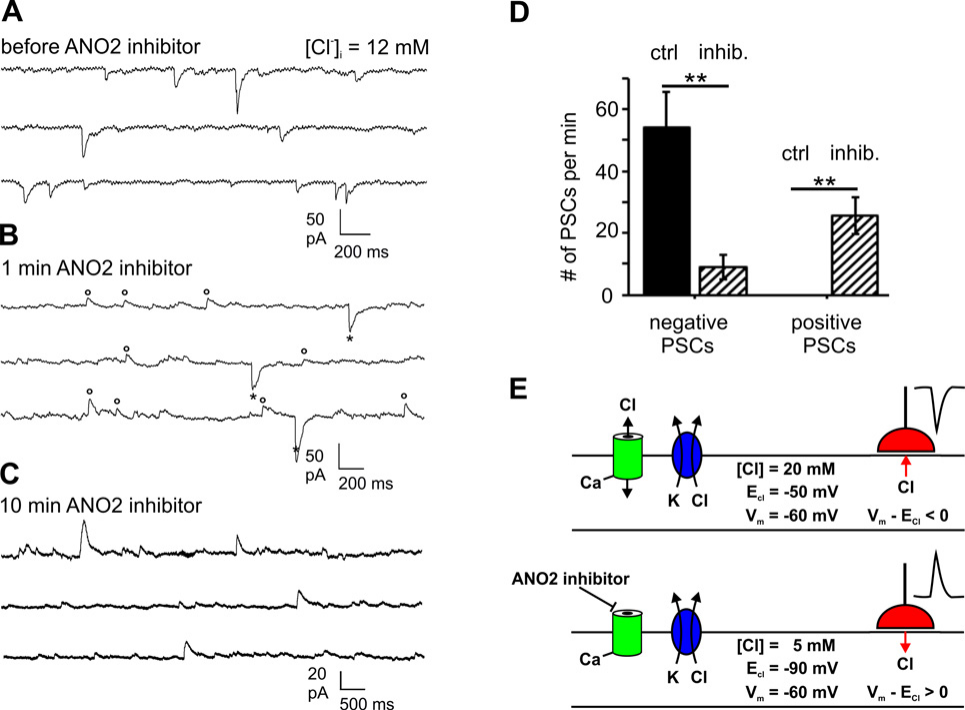
Reversal of GABAergic postsynaptic currents by the ANO2 inhibitor. **(A)** At a Cl^-^ concentration of 12 mM in the recording pipette, GABAergic postsynaptic currents were negative (Cl^-^ efflux), indicating that postsynaptic E_Cl_ is less negative than V_hold_. **(B)** Shortly after applying 5 μM ANO2 inhibitor, positive currents appear *(circles)* as some synapses experience a decline of postsynaptic [Cl^-^]_i_, while others still have high Cl^-^ *(asterisks)*. **(C)** During the continued presence of the ANO2 inhibitor, virtually all postsynaptic currents reverse to positive polarity (Cl^-^ influx) indicating that GABAergic synapses experience an E_Cl_ more negative than V_hold_. **(D)** The collected data from 12 Purkinje cells at [Cl^-^]_i_ = 12 mM and V_hold_ = -60 mV demonstrate the polarity reversal of postsynaptic currents (PSCs) actuated by the ANO2 inhibitor. **(E)** Schematic representation of an hypothesis for the 12 mM [Cl^-^]_i_ experiment. In the absence of the ANO2 inhibitor *(upper scheme)*, the basal activity of ANO2 channels *(green)* provides a Cl^-^ conductance in the dendritic membrane. ANO2 contributes to the Cl^—^transport machinery, whose various pathways are represented by the K^+^/Cl^—^cotransporter KCC2 *(blue)*. Together the Cl^-^ pathways stabilize a slightly elevated level of [Cl^-^]_i_ which results in a negative driving force (V_m_—E_Cl_ < 0) for Cl^-^ currents through GABA_A_ receptors in GABAergic synapses *(red)*. In this situation, Cl^-^ currents are outwardly directed and cause negative postsynaptic currents. Application of the ANO2 inhibitor *(lower scheme)* reduces the Cl^-^ conductance. This causes a polarity reversal of the Cl^-^ driving force, as the balance shifts towards Cl^-^ extrusion, causing local [Cl^-^]_i_ to decrease. This hypothesis provides a qualitative concept for the role of ANO2 channels in the inversion of postsynaptic currents that is depicted in panels A to C. The proximity of Cl^—^transport pathways and GABAergic synapses, as well as the occurrence of local Cl^-.^gradients within dendritic segments, are inspired by the model for GABA_A_-receptor-mediated Cl^-^ gradients in extended dendritic trees proposed by Jedlicka et al. (2011) [88].

If ANO2 modulates IPSCs in GABAergic synapses by changing postsynaptic [Cl^-^]_i_, as suggested by the findings of Satoh et al. (2013) [31], the polarity and amplitude of IPSCs should depend on the activity of ANO2. We tested this assumption under conditions where a *reversal* of IPSC polarity may report an increase of [Cl^-^]_i_ at synaptic sites. We used 12 mM [Cl^-^]_i_ (E_Cl_ near -62 mV) in the pipette solution and clamped the holding-voltage to -60 mV. The rationale was to record GABAergic signals with E_Cl_ set close to V_hold_, so that any driving force for Cl^-^ could only result from local changes of [Cl^-^]_i_ near the GABAergic synapses. Under these conditions, the postsynaptic currents had negative polarity indicating that E_Cl_ at the synaptic sites was more positive than V_hold_ and that GABA_A_ receptors conducted Cl^-^ efflux (Fig 6A). Current amplitudes were small as a consequence of the small driving force for chloride ions (V_m—_E_Cl_ < 0). Shortly after perfusion of the ANO2 inhibitor, both positive and negative signals were recorded (Fig 6B) and, upon continuous application of the inhibitor, most postsynaptic signals were inverted to positive polarity (Fig 6C). This effect of the inhibitor was observed at various times after the start of the experiment, but the polarity switch was never observed without the inhibitor. An analysis of signal traces from 9 Purkinje cells at 12 mM [Cl^-^]_i_ showed that the ANO2 inhibitor decreased the incidence of negative postsynaptic signals from 54.0 ± 11.6 min^-1^ to 9.0 ± 4.2 min^-1^, while the incidence of positive signals increased from zero to 25.7 ± 6.0 min^-1^ (Fig 6D). Thus, application of the ANO2 inhibitor triggered a shift of the local E_Cl_ from a value more positive than V_hold_ to a value more negative than V_hold_, caused by a drop of local [Cl^-^]_i_ at the synaptic sites. This result is consistent with a modulatory effect of ANO2 on local [Cl^-^]_i_ under these conditions.

It appears that, at 12 mM [Cl^-^]_i_ in the pipette solution, the various chloride pathways present in the Purkinje cell dendrite uphold a chloride level that supports Cl^-^ efflux through GABA_A_ receptors (Fig 6E, *upper scheme*). If, however, the ANO2 Cl^-^ conductance is blocked by *T16Ainh-A01* over a prolonged period of time, the balance between Cl^-^ uptake and Cl^-^ extrusion seems to change. Local [Cl^-^]_i_ decreases and promotes Cl^-^ influx through GABA_A_ receptors (Fig 6E, *lower scheme*). This interpretation of the data presented in Fig 6 suggests that, even without experimentally induced climbing-fiber activation, ANO2 channels have a sufficient basal activity to influence local [Cl^-^]_i_ homeostasis near the synaptic sites. This may be the consequence of some excitatory input, and hence Ca^2+^ influx, in the slice preparation. These experiments suggest that ANO2 channels are a component of the transport system that regulates postsynaptic [Cl^-^]_i_ in the Purkinje-cell dendritic tree. As a Ca^2+^-gated channel, ANO2 is expected to exert its effect most efficiently near sites of Ca^2+^ entry. We assume that the dendritic spines are the main source of Ca^2+^ entry [53], and that Ca^2+^ signals are strongest in the distal dendrites. This may be due to the larger surface-to-volume ratio and a higher density of voltage-gated Ca^2+^ channels in distal dendrites [54]. ANO2 in this region may contribute to *DDI* more efficiently than in the soma and proximal dendrite (see Discussion).

Taken together, the effects of the ANO2 inhibitor consistently demonstrate that ANO2 channels operate in the Purkinje cell plasma membrane. ANO2 appears to affect the regulation of local [Cl^-^]_i_ levels in the Purkinje cell dendritic tree. Under physiological low chloride conditions, ANO2 channels mediate a Ca^2+^-dependent Cl^-^ uptake into the dendrite which causes an increase of local [Cl^-^]_i_ and, hence, a reduced driving force for Cl^-^ entry through GABA_A_ channels. Thus, ANO2 channels appear to mediate depolarization-induced depression of inhibitory transmission, *DDI*, as described by Satoh et al. (2013) [31].

## Discussion

### ANO1 and ANO2 are expressed in the cerebellar cortex

We have examined the questions whether ANO1 and ANO2 proteins form Ca^2+^-activated Cl^-^ channels in the cerebellar cortex, and whether these channels contribute to the *DDI* form of synaptic plasticity. PCR experiments and immunoblots showed that both ANO1 and ANO2 are expressed. Immunosignals indicated expression of both proteins in Purkinje cells, but ANO1 also in basket, stellate and Golgi cells. We identified the splice variant ANO1*ac*, a variant previously shown to possess particularly high Ca^2+^ sensitivity. The EC_50_ for channel activation by Ca^2+^ at -40 mV was reported to be 0.13 μM for ANO1*ac* and 0.63 μM for ANO1*abc* [5]. Segment *a* sensitizes ANO1 to Ca^2+^ [55], and segment *c* stabilizes the open state of the channel [56]. Immunostaining of ANO1 and ANO2 in the cerebellum required the preparation of cryosections without prefixation of the brain, a protocol known to increase detection sensitivity. Total protein extracts from tissue lysates did not produce anoctamin-specific signals in immunoblots, as reported earlier for ANO2 [8]. It was necessary to remove nuclei and soluble proteins and to enrich membrane proteins. These observations point to a relatively low expression level of ANO2 in the cerebellum, consistent with our RT-PCR analysis and immunostaining. The ANO1*ac* protein is expressed in all GABAergic neurons of the cerebellar cortex. Its function in these cells remains to be elucidated in future studies. In Purkinje cells, ANO1 appears to be restricted to the cell body and is hardly detectable in the dendritic tree. Possibly, the channels provide a Ca^2+^-dependent component to cell volume control, as was suggested for ANO1 in epithelial cells [57]. It is, however, unlikely that ANO1*ac* channels contribute significantly to *DDI*. Because the application of 5 μM ANO2 inhibitor, as well as the ablation of the Ano2 gene, completely removed *DDI*, our data strongly suggest that ANO2 channels mediate *DDI*.

The evidence for an involvement of ANO2 in ionic plasticity presented here is based on the comparison between wild-type Ano2^+/+^ mice and Ano2^-/-^ knockout mice. The availability of the Ano2^-/-^ mouse line [8] strengthens the validity of the data, which would otherwise have to rely on the specificity of ANO2 antisera and the ANO2 inhibitor. However, the lack of dendritic ANO2 immunosignals in Ano2^-/-^ Purkinje cells, together with the resistance of IPSCs to CF-stimulation in the knockout, strongly corroborate the hypothesis that ANO2 is necessary to trigger *DDI*. Nevertheless, it appears striking that the impact of 5 μM ANO2 inhibitor on IPSCs is strong enough to mimic the effect of an ANO2 knockout, considering that the blocker reduces ANO2 currents in HEK293 cells by only 27% at that concentration. This observation can be interpreted as a cumulative effect that developed during the extended presence of the blocker. The ANO2 inhibitor was applied for several minutes in our experiments. The continuously reduced dendritic Cl^-^ permeability during this time is expected to result in a progressive change of dendritic [Cl^-^]_i_. Thus, temporal aspects have to be considered for the evaluation of the ANO2 inhibitor data. This is illustrated by Fig 6 where the inversion of postsynaptic current polarity is seen to develop with time.

Our data indicate that ANO2 channels are active even without climbing fiber activation and despite the presence of EGTA in our pipette solution. This is surprising, considering that Purkinje cells have a particularly high Ca^2+^ buffer capacity [58] and that the baseline Ca^2+^ concentration is expected to be low [47,59]. It indicates that, in our preparation, local Ca^2+^ fluctuations in the dendrites are sufficient to induce some degree of ANO2 activity. The isoform *B* of ANO2, which is expressed in Purkinje cells, is half-maximally activated at 1.33 μM Ca^2+^ [6]. Thus, channel activity, detected through effects of the channel blocker in the present study, may reflect Ca^2+^ concentrations of ~1 μM at the expression sites of ANO2. This points to a proximity of ANO2 channels and the sites of synaptic Ca^2+^-signal generation in Purkinje-cell, the dendritic spines. It appears that the dendritic ANO2 channels respond to the Ca^2+^ concentration in the spines, and that they influence the local Cl^-^ concentration inside the spines. The range of Ca^2+^-concentrations in which cerebellar ANO2 channels operate (1–10 μM) [9,11,38] corresponds well to the dynamic range of Ca^2+^ transients measured in Purkinje cells. Ca^2+^ concentrations of 0.5 to 4 μM induce long-term depression upon activation of climbing fibers [59]. These matching Ca^2+^ ranges suggest that the open probability of dendritic ANO2 channels increases when excitatory synaptic inputs generate dendritic calcium signals through activation of voltage-gated Ca^2+^ channels and Ca^2+^ release (reviewed by [54]). ANO2 appears to be a Ca^2+^-dependent element of chloride homeostasis in Purkinje cell dendritic spines. In concert with the chloride exporter KCC2 [60,61], the channels appear to set local levels of [Cl^-^]_i_ and, hence, to co-determine the efficacy of GABAergic transmission. A contribution of the chloride importer NKCC1 was suggested [31] because 50 μM bumetanide reduced an initial, transient phase of *DDI* that was observed in that study. However, expression of NKCC1 in Purkinje cells is not established; several studies report the absence of NKCC1 mRNA from Purkinje cells in the adult cerebellum [62–64].

### ANO2 channels and synaptic plasticity in the cerebellum

The formation of procedural memory, in particular the acquisition of motor skills, involves the cerebellum as central element in the preparation, the execution, the fine adjustment, and in the learning of movement. The characteristically uniform microcircuits in the cerebellar cortex and the deep nuclei constitute the hub of signal processing for these tasks [65–68]. It is well documented that plasticity of inhibition in GABAergic synapses is a major factor in shaping the output pattern of the cerebellar cortex [69–71]. The sites of plasticity are the synapses formed by inhibitory interneurons on the Purkinje cell dendrites and somata. Various distinct modes of synaptic plasticity control the efficacy of inhibitory transmission in these synapses: *rebound potentiation (RP)*, a postsynaptic Ca^2+^/ CaMKII-mediated process that transiently enhances the responsiveness of GABA_A_ receptors upon depolarization [72–75]; *depolarisation-induced potentiation of inhibition (DPI)*, a delayed, long-lasting increase of GABAergic transmission mediated by presynaptic NMDA-receptors [76]; *depolarization-induced suppression of inhibition (DSI)*, a reduction of IPSPs for ~20 s induced by retrograde inhibition of GABAergic synapses through endocannabinoids [37,77–79]; *depolarization-induced depression of inhibition (DDI)*, a decrease of IPSC amplitudes as a consequence of increasing postsynaptic Cl^-^ concentration, mediated by dendritic Ca^2+^-activated Cl^-^ channels and, possibly, Cl^-^ transporters [31]. Taken together, these modes of synaptic plasticity reflect the relevance of modulatory processes for GABAergic inhibition of Purkinje cell activity.

The Ca^2+^-dependent Cl^-^ conductance observed in Purkinje cells [77] was previously shown to be involved in the generation of *DDI* [31]. *DDI* changed the amplitudes of IPSCs that were evoked by stimulation of interneurons in the molecular layer and mediated by GABAergic synapses. The basic observation was that the postsynaptic currents transiently changed polarity from outward to inward (*i*.*e*. from inhibitory to excitatory), when a series of depolarizing pulses was applied to the Purkinje cells. Even after partial recovery and restoration of outward polarity, the IPSC amplitudes remained small for over 20 min. Satoh et al. (2013) [31] presented evidence that *DDI* was caused by a Ca^2+^-dependent rise of [Cl^-^]_i_ on the postsynaptic side (in the Purkinje cell dendrite), resulting in a decreased driving force for Cl^-^ influx through GABA_A_ receptors. As such, *DDI* would be an example of ionic plasticity [80–83], an altered synaptic transmission, brought about not by GABA_A_ receptor regulation but because of a changing driving force for Cl^-^ flux.

In the present study, changes in [Cl^-^]_i_ were inferred from the effect of the ANO2 blocker on the amplitude of postsynaptic currents in GABAergic synapses. The interpretation of this indirect read-out is based on the assumption that the amplitudes of GABA-induced postsynaptic currents depend on [Cl^-^]_i_ and mirror any change of [Cl^-^]_i_. A decline of IPSC amplitude may, however, also be caused by changes in the neurotransmission process or by changes in the passive electrical properties of the dendrite. Such an electrical effect was proposed for ANO2 activation near excitatory synapses in hippocampal pyramidal neurons [27]. However, changes of GABA transmission in Purkinje cells following CF-activation occur on much slower time scales than used in this study [84] and require phosphorylation and recruitment of new receptor subunits. Moreover, it is unlikely that the decrease of IPSC amplitudes after CF-activation results from passive electrical effects like the introduction of a shunt conductance. This can be inferred from the effects of CF-activation on EPSPs at parallel-fiber synapses on Purkinje-cell dendrites. Time course and amplitude of EPSPS are not altered by CF-activation, provided that retrograde inhibition is blocked by a cannabinoid-receptor antagonist [85]. Thus, within the context of *DDI*, it is reasonable to assume that decrease and inversion of IPSCs observed here result from changes in [Cl^-^]_i_. Nevertheless, it would be desirable to directly measure changes of [Cl^-^]_i_ by chloride imaging, a method that was successfully applied to Purkinje cell dendrites using two photon excitation of the fluorescent dye MQAE [86]. This is technically demanding but may be a suitable approach to assess the contribution of ANO2 channels to [Cl^-^]_i_ regulation quantitatively.

The neuronal chloride homeostasis is mediated to a large extent by cation-chloride cotransporters, in particular by KCC2 and, in some neurons, by NKCC1 [87]. Chloride channels can, however, transiently change [Cl^-^]_i_, especially within the restricted space of distal dendrites [88–90]. This was studied in detail for the dendrites of hippocampal pyramidal neurons, where, as a consequence of intense of GABA_A_ receptor activity, [Cl^-^]_i_ increases and depolarizes E_Cl_ [91–93]. This GABA-induced Cl^-^ uptake can overcome the intrinsic Cl^-^ extrusion mechanism in parts of the dendrite with high density of inhibitory synapses. It can change E_Cl_ locally, and can hence cause a spatially restricted attenuation—or even inversion—of GABA effects [81]. In a similar way, ANO2 channels may affect [Cl^-^]_i_ and attenuate GABAergic input in the Purkinje cell dendrite, when activated by Ca^2+^ near excitatory synapses. In contrast to the homeostatic regulation of [Cl^-^]_i_ by cation-chloride cotransporters, the effect of ion channels on dendritic E_Cl_ ceases after Cl^-^ channels close and Cl^-^ extrusion prevails. Satoh et al. [31] reported, that Purkinje cells largely recovered from *DDI* within 2–3 minutes, but that inhibition was still slightly attenuated 20 min after *DDI* induction. If ANO2 channels mediate *DDI*, this slow time course of recovery reflects three processes following the end of CF-activation: return of dendritic Ca^2+^ levels to sub-micromolar levels, closing of ANO2 channels, and Cl^-^ extrusion from the dendrite. Ca^2+^-imaging studies during CF-activation indicated that the Ca^2+^ decline proceeds within less than a second [58], and the open probability of ANO2 follows changes in Ca^2+^ concentration within a fraction of a second [11]. This suggests that the recovery dynamics of *DDI* are determined by the net rate of Cl^-^ extrusion. This rate may be determined by Cl^-^ imaging in future studies.

In conclusion, we have analyzed the expression of ANO1 and ANO2 Ca^2+^-activated Cl^-^ channels in the cerebellar cortex. While ANO1 is expressed in inhibitory interneurons and in the somata of Purkinje cells, ANO2 is absent from inhibitory interneurons but specifically targeted to the Purkinje cell dendritic tree. ANO2 channels have previously been studied in dendritic signal processing of olfactory receptor neurons [6–10] and hippocampal pyramidal neurons [27] as well as in the pre-synaptic terminals of rod photoreceptors [14,15,94]. Here we provide evidence that dendritic ANO2 channels co-determine local chloride concentrations near GABAergic synapses. ANO2 channels appear to mediate *DDI*, a Ca^2+^-dependent form of ionic plasticity that attenuates GABAergic inhibition in cerebellar Purkinje cells.
